# Effect of integrated reproductive health lesson materials in a problem-based pedagogy on soft skills for safe sexual behaviour among adolescents: A school-based randomized controlled trial in Tanzania

**DOI:** 10.1371/journal.pone.0263431

**Published:** 2022-02-22

**Authors:** Walter C. Millanzi, Stephen M. Kibusi, Kalafunja M. Osaki

**Affiliations:** 1 Department of Nursing Management and Education, School of Nursing and Public Health, The University of Dodoma, Dodoma, Tanzania; 2 Department of Public Health, School of Nursing and Public Health, The University of Dodoma, Dodoma, Tanzania; 3 Centre for Pedagogy, Education, Development, Writing and Editing Documents, St. Augustine University of Tanzania, Mwanza, Tanzania; University of Macau, MACAO

## Abstract

**Background:**

Adolescents are currently becoming sexually active before their 18th birthday during which they have to battle with unsafe sexual behaviours, teenage pregnancies, sexually transmitted infections (STIs), and school dropouts. The trend is linked with low soft skills (self-esteem and assertiveness skills) for them to make informed, reasoned, and responsible decisions over sexual activities. This study designed and tested the effect of integrated reproductive health (RH) lesson materials in a problem-based pedagogy (PBP) to enhance soft skills for safe sexual behaviour among adolescents in Tanzania.

**Methods:**

A double-blinded clustered randomized controlled trial was conducted between September 2019 and September 2020 among 660 randomly selected adolescents. A Sexual-risk Behaviour Beliefs and Self-esteem Scale adopted from previous studies measured soft skills for safe sexual behaviour. A descriptive statistical analysis was performed by using the statistical analysis software programme version 9.4. The effect of the intervention was determined using Linear Mixed Model set at α error probability = 5% significance level (95% confidence interval) and a β error probability = 0.80.

**Findings:**

Adolescents’ mean age was 15±1.869 with 57.5% females. The end-line findings indicated that the coefficient of soft skills was significantly higher among adolescents in the hybrid PBP (β=9.0986, p<0.01; 95%CI: 4.7772, 14.2311) and pure PBP (β =8.7114, p<0.01; 95%CI: 3.9990, 10.1208) than in the control group. The retention rate of soft skills was still significantly higher at 3-months follow-up (β=2.0044; p<0.01; 95%CI: 1.0234, 4.1182) and at 6-months follow-up (β=1.9803; p<0.01; 95%CI: 0.8399, 3.1099) compared to the baseline and immediate post-intervention assessments.

**Conclusion:**

The intervention substantially enhanced soft skills for safe sexual behaviour among adolescents of both sex. Despite the fact that scores for soft skills varied across the study timelines, adolescents demonstrated significant intentions to abstain from sexual intercourse, delay sexual relationships, negotiate condom use, and withstand sexual coercions. The PBP may need to be incorporated in ordinary level secondary school curricula as a formal guide to teachers and or health workers to optimally prepare adolescents for their healthy adulthood.

## Introduction

Adolescents represent a large cohort that needs to be developed with safe, good, and age-appropriate personal characters, identity, and social responsibilities for healthy adulthood [1]. Studies have defined adolescence as a transition period from childhood to adulthood characterized by biological, pubertal, and neurobehavioural changes among children [2]. The stage characterizes them not only to be attention seekers but also to demonstrate reckless behaviours including drug abuse, robbery, theft, engaging in nightclubs, sleeping outside their home, unsafe sexual behaviours, and disputes with their parents, peers, and teachers at schools [3]. It is a period where adolescents experience outbursts of sexual emotions, mood disruptions change in social responsibilities that need close parental guidance, monitoring, and support towards their life potentials.

Unsafe sexual behaviours among adolescents aged between 10 and 19 years for example have become a prevailing problem around the globe [4]. Unsafe sexual behaviours here are defined as such manners as early initiation sexual behaviours, pre-marital sexual intercourse, incorrect and inconsistent usage of contraceptives, having multiple sexual partners, frequent sexual intercourse, drug abuse before, or while having sexual activity, and or engaging in sexual intercourses for money or materials gain [5]. On contrary, safe sexual behaviours are one’s ability to abstain, report sexual harassment/abuse/debuts, decide optional activity over sexual temptations, and periodic seeking of health care services.

Other safe sexual behaviours include avoiding drug abuse, avoiding multiple sexual partners, coerced sexual intercourse, or doing sexual intercourse for material gain/business, and using condoms appropriately and consistently [6]. Christopher *et al*., [7] unfold that unsafe sexual behaviours among adolescents are probably very obvious with poor self-regulation of sexual emotion and behaviour at an early age. It has been exposed that most adolescents have less soft skills to decide or negotiate safer sexual behaviours. Soft skills are defined here as a set of non-academic competencies, behaviours, attitudes, and personal qualities, which enable people to effectively navigate their environment, work well with others, perform well and achieve their goals [8].

In the current study, soft skills are defined as the adolescents’ self-esteem, assertiveness skills, intention to abstain or be faithful, pre-emptively recognizing forced sexual relationships, negotiation skills, report, and refusal strategies of unsafe sexual activities. Adolescents who have adequate soft skills are expected to make conscious, informed, and reasoned decisions over their sexual activities [8]. They are believed to be able to demonstrate reasoned judgment and intentions to opt for alternative strategies to sexual temptations and coercion [7]. Timely and age-appropriate sexual and reproductive health (SRH) information and education among adolescents are closely linked with not only SRH knowledge, development of appropriate personal characters, identity, social roles, and responsibilities, but also soft skills for safe sexual behaviours [9].

There are numerous initiatives in place to advocate adolescents’ sexual health needs and rights including the development of various health policies and SRH guidelines which are implemented across the world [10, 11]. Interventions such as but not limited to building boarding schools, enrolment of students to stay in school hostels, health policy, sexual education clubs, large scale reproductive and family planning methods campaigns, projects, and sexual health education training among teachers; are being implemented to address unsafe sexual behaviours among adolescents [12, 13].

The National School Health Programs provides adolescents with many health care services such as SRH information and its associated services and counselling support to address their problems [13]. The Ministry of Education, Science and Technology in Tanzania has also brought sexual education and STIs and Human Immunodeficiency Virus (HIV) education into the national school curriculum [14–16]. Amid all efforts, educational and health stakeholders claim that there are inadequate innovative educational efforts that promote soft skills among the cohort of adolescents. Different reports on SRH education in Tanzania [UNESCO 17, 18)], Human Right Watch [19], Mpondo [20], Kalolo and Kibusi [21], and Mkumbo [22] suggest that adolescents in Tanzania are not well empowered with the necessary soft skills for safe sexual behaviour change.

The problem is linked either to the ways sexual health information and education are facilitated in schools, the attitude and skills of teachers and healthcare workers towards facilitating SRH learning to adolescents, or something might be missing in the curricula. However, scholars and health and education stakeholders argue that the existing SRH guidelines and materials seem to lack a formal pedagogical guide to help teachers and or health workers in facilitating SRH learning to adolescents in schools [23]. The existing pedagogies (lectures, discussions, or demonstrations) of facilitating SRH education among adolescents have been reported to make health workers, religious leaders, parents, guardians, and schoolteachers experience challenges in assisting them to develop age-appropriate SRH knowledge and or soft skills for self-control against sexual temptations, harassment, and pressure when they resort to using didactic pedagogies.

Although Mkumbo [22] has exposed that teachers and or health workers have a positive attitude towards facilitating SRH learning among adolescents. However, they report experiencing some difficulties when they try to facilitate the social and physiological part of it by using lectures, discussions, and or demonstrations. Haruna *et al*., [24] added that teachers and or health workers who are invited to facilitate SRH learning to adolescents in schools focus more on biological aspects such as mammal and human reproductive systems by using conventional pedagogies because it is easy to implement. However, they feel shy and experience pedagogical difficulties in facilitating the psychological part of the materials such as ways to control sexual outbursts, mood disruptions, and or how to deal with coerced sexual relationships and sexual intercourses using conventional pedagogies.

Scholars encourage basic education to adopt and implement participatory pedagogies in facilitating SRH learning among adolescents. Chavula *et al*., [25] argued that if a comprehensive SRH education is facilitated by using participatory and collaborative pedagogies, the interaction and communication about sensitive reproductive topics among teachers, healthcare workers, and other facilitators become very easy. Problem-based pedagogy (PBP) rooted in the social constructivist theory of learning is said to be one of the participatory pedagogies in education in both, well and resource-poor settings [26–28]. The PBP is an instructional strategy that uses real-life or hypothetical problems as motivators of learning activities and a stimulator of new ideas among learners. The pedagogy can be implemented in two forms including a pure PBP and hybrid PBP.

Pure PBP is characterized by the use and introduction of real-world or hypothetical problems to students without any other forms of pedagogies such as lecture while the hybrid PBP uses both, a real-world problem and some forms of other pedagogies. The PBP is characterized by introducing problems first then the learning process among learners who work collaboratively in small groups (5 to 8 members per group) to find the sustainable solution to the assigned problems [29]. In contrast to conventional pedagogies such as lecture-based pedagogy (LBP), the PBP is believed to hold some potentials of empowering learners with the ability to research and find a viable solution to the defined ill-structured, or real-life problems [29]. Through the PBP, students attempt to resolve ill-structured real-life problems by using the learners’ prior knowledge and experiences.

The pedagogy commenced around the 1950s at McMaster University, Canada in response to the didactic approach which was seen to be less effective in shaping the learners’ behaviours [30]. The pedagogy has come to the forefront of discussions in curricula and classroom reform among educators to prepare graduates who can demonstrate critical thinking and problem-solving skills [30]. According to Jonassen and Hung [31] and Barrows [29], PBP is effective in a diversity of teacher-training programmes, and higher school and elementary, middle, and low-income students. The PBP is now implemented in a variety of schools including medicine, business administration, engineering studies, leadership education, architecture, teacher education, nursing, chemical engineering, and social work disciplines.

Although very few, available comparative scholarly works have demonstrated pieces of evidence of the effect of PBP pedagogy over the LBP in secondary school students’ learning behaviours by influencing them to take responsibility for their learning with minimal support and guidance from facilitators. The work of Keziah [32] for example, has shown that PBP enhances behaviour change in students’ learning habits by motivating them to learn biology (M = 1268.50; SD = 130.63) more than the LBP did (M = 818.00; SD = 135.89). On the contrary, Karimi *et al*. [33] explored the effect of PBP on improving health behaviours among girls. They found that the total girls’ health literacy significantly improved (p<0.001) with higher mean scores after intervention than girls in the control group did.

Despite its academic potentials among learners, the PBP is almost not known and controversial whether it can be used as a guide to teachers and or health workers in facilitating SRH learning to adolescents. It is not clear if the use of integrated RH lesson materials in a PBP can demonstrate potential effects in enhancing soft skills for safe sexual behaviours among ordinary level secondary schools in Tanzania. This study, therefore, aimed at examining the effect of integrated RH lesson materials in a PBP in enhancing soft skills for safe sexual behaviour among adolescents in Tanzania.

## Materials and methods

Ethics and methods were performed per the University of Dodoma (UDOM) guidelines and regulations [34]. Written informed consent was obtained from all participants who were above 18 years and teachers and or legally acceptable representatives consented in writing for minors (below 18 years of age) as one of the criteria to participate in the study. The trial was registered by Pan Africa (PACTR) on 02/09/2020 with the number PACTR202009656160779.

### Study location

The study was conducted in ordinary-level secondary schools in Dodoma and Lindi regions, Tanzania.

### Study design

A Clustered Randomized Controlled Trial (RCT) was adopted in this study.

### Study arms

The study arms in this work were meant by the number of research groups involved in interventions (It was equivalent to the term “sub-study”). Two research arms were involved during the randomization procedure including the PBP (intervention) including the pure PBP and hybrid PBP and Lecture-based pedagogy (LBP) (control). The aim of studying three arms was to discriminate the interventions’ effects on adolescents’ soft skills thus strengthening the validity and credibility of the findings. Two (2) intervention arms were implemented in the nature of PBP (pure and hybrid PBP) of which, adolescents assigned in the first intervention arm (pure PBP) received integrated RH lesson materials by pure PBP (constituted by an integrated RH lesson materials + PBP+ without LBP).

The second intervention arm received the same RH lesson materials via hybrid PBP (constituted by an integrated RH lesson materials + PBP + LBP). The pure PBP did not integrate any form of lecture (adolescents were assigned problems in a scenario and figurative language and facilitated to solve them on their own) while the hybrid employed mini-lecture to provide some descriptions of the scenario alongside their procedure to solve them. Moreover, adolescents assigned in the control arm received the standard RH lesson materials through the LBP only (constituted by standard RH lesson materials + LBP).

### Intervention

The intervention was conducted in a parallel design in 12 well-separated ordinary level secondary schools in Lindi and Dodoma regions, Tanzania. Public ordinary level secondary schools were involved in the study where schools were sampled from each of the four districts and were randomly allocated to either pure PBP, hybrid PBP, or LBP. As shown in Table 1, the interventions employed group education that aimed at empowering adolescents to develop soft skills for safe sexual behaviours. The RH lesson materials alongside its associated teaching and learning experiences adopted designs and pedagogies from the existing Tanzanian Biology curricula and syllabus [15] and previous studies [28, 35].

**Table 1:** Descriptions of interventions per study arms.

	Pure PBP	Hybrid PBP	LBP
**Content**	Theoretical and practical concepts of SRH lesson materials	Theoretical and practical concepts of SRH lesson materials	Theoretical and practical concepts of SRH lesson materials
**Facilitator**	Trained research assistants in all four axes who also had expertise in RH	Trained research assistants in all four axes who also had expertise in RH	Trained research assistants in all four axes who also had expertise in RH
**Learners**	Ordinary level secondary school adolescents	Ordinary level secondary school adolescents	Ordinary level secondary school adolescents
**Delivery mode**	Face-to-face	Face-to-face	Face-to-face
**Pedagogy**	PBP pedagogy + RH lesson materials	PBP pedagogy + LBP + RH lesson materials	LBP + RH lesson materials
**Session commencement**	Sessions commenced with greetings followed by the presentation of a real-life or hypothesized problem in ill-structured scenarios and puzzling pictures as a learning catalyst per session	Sessions commenced with greetings followed by the presentation of a real-life or hypothesized problem in ill-structured scenarios and puzzling pictures as a learning catalyst per session	Sessions commenced with greetings followed by a facilitator giving descriptions of the topic of a particular session then questions and answers
**Dose**	Four sessions	Four sessions	Four sessions
**Frequency**	One session per day with a maximum of two sessions a week for four weeks	One session per day with a maximum of two sessions a week for four weeks	One session per day with a maximum of two sessions a week for four weeks
**Time per session**	Ranging from 30 minutes to 90 minutes depending on the amount of content, teaching, and learning activities	Ranging from 30 minutes to 90 minutes depending on the amount of content, teaching, and learning activities	Ranging from 30 minutes to 90 minutes depending on the amount of content, teaching, and learning activities
**Timing**	Sessions were conducted during morning times (half-day sessions) after some negotiations made with the head of the respective schools	Sessions were conducted during morning times (half-day sessions) after some negotiations made with the head of the respective schools	Sessions were conducted during morning times (half-day sessions) after some negotiations made with the head of the respective schools
**Sitting plan**	The classrooms were set for adolescents to sit in a round style to promote eye contact during presentations	The classrooms were set for adolescents to sit in a round style to promote eye contact during presentations	The classrooms were set for adolescents to sit facing the front of the class where a facilitator is
**Group formation**	Adolescents had to learn the RH contents in groups of 5-8 members	Adolescents had to learn the RH contents in groups of 5-8 members	Adolescents learned the RH contents via the facilitator-led method (no groups)
**Self-study**	Adolescents were given a minimum of 30 minutes for self-study about the problem to explore and identify potential solutions to solve it	Adolescents were given a minimum of 30 minutes for self-study about the problem to explore and identify potential solutions to solve it	Adolescents were not given a time for self-study as they received RH lesson materials through the facilitator-led method
**Time for group works**	Thirty minutes were then added for adolescents to share and discuss their works within their groups of 5 to 8 members	Thirty minutes were then added for adolescents to share and discuss their works within their groups of 5 to 8 members	There was no time for group works rather than questions and answers that were addressed among adolescents and a facilitator during the session
**Assignments**	Extra time was given to adolescents to continue analysing the problem and identifying appropriate solutions to address them as a take-home activity to be shared in the next session	Extra time was given to adolescents to continue analysing the problem and identifying appropriate solutions to address them as a take-home activity to be shared in the next session	There was no extra time for group works rather than questions and answers that were addressed among adolescents and a facilitator during the session
**Class presentations**	Adolescents in their groups had to present their works in the entire class, defend, and address any queries from their colleagues before they were peer-rated.	Adolescents in their groups had to present their works in the entire class, defend, and address any queries from their colleagues before they were peer-rated.	There were no class presentations because there were no group works or assignments
**Adolescents’ evaluation**	All adolescents received the evaluation via pre-post-tests	All adolescents received the evaluation via pre-post-tests	All adolescents received the evaluation via pre-post-tests
**Mode of in-class adolescents’ evaluation**	Peer-rating + facilitator-rating + correction of misinterpreted concepts	Peer-rating + facilitator-rating + correction of misinterpreted concepts	Facilitator-rating
**Mode of end of session evaluation**	2 to 5 adolescents were randomly selected to share their experiences about the session including the teaching and learning styles	2 to 5 adolescents were randomly selected to share their experiences about the session including the teaching and learning styles	There was no end of session evaluation

**Source:** Study Plan (2020).

The interventions were delivered by twelve trained research assistants who also had expertise in RH. They did so by using Biology and Civics clubs. One trained assistant was then assigned per study location (One ordinary level secondary school) to facilitate the intervention throughout the four weeks and was responsible to implement all the themes as prescribed in the integrated RH lesson materials. That is, a total of 142 adolescents in the pure PBP group, 188 adolescents in the hybrid PBP group, and 317 adolescents in an LBP group a standard RH lesson materials. More descriptions about interventions per research arms are shown in Table 1. There were no important changes to the methods of delivering the intervention after the commencement of the study.

### Criteria for discontinuing or modifying allocated intervention

Adolescents with prolonged engagement in the intervention with regular class attendances continued to participate in the study. Regular class attendances among adolescents were defined as adherence to day-to-day participation of class sessions and learning activities as scheduled throughout the interventions. Adolescents who dropped out without or with notification to the researcher or assistants at any point, were discontinued from the study and thus, their information was not analysed. Adolescents who requested to disjoin the study were discontinued.

### Strategies to improve adherence to the intervention

This study was conducted in line with the schools’ academic schedules. Considerations were taken into account not to involve any form of invasive procedures among adolescents. Adolescents’ code numbers were used as their identifiers (Names) throughout the study duration. Attendances were taken at both the beginning and end of each session of the intervention. After each day’s session, adolescents were assigned simple works to do as homework to be shared in the next session.

Examples of simple works adolescents were assigned included preparing storytelling about sexual coercions events encountered in life, finding real-life strategies to solve a presented problem in a scenario, finding a more relevant source of information over the provided ones, and or attaching meaning of the RH lesson materials in own body changes. One of the secondary school teachers of each study centre was involved to help facilitate adherence and response rate of the adolescents.

### Study population

The target population was school-going adolescents aged between 10 and 19 years old in Tanzania. Contrary to Demographic Health Surveys (DHS) and other studies, data more often describe adolescents aged between 15 to 19 years or included in young adults (15 to 24 years) than younger adolescents (10 to 14 years). This study intended to investigate the full range of 660 adolescents between 12 to 19 years in Tanzania’s mainland. Adolescents between groups were matched in their ages, and years to ensure their demographic characteristic similarities before the commencement of the study. Written informed consent was obtained from all participants and written informed consent was obtained from parents/teachers or legally acceptable representatives for minors (below 18 years of age) as one of the criteria to participate in the study.

### Eligibility criteria

**Fig 1:**
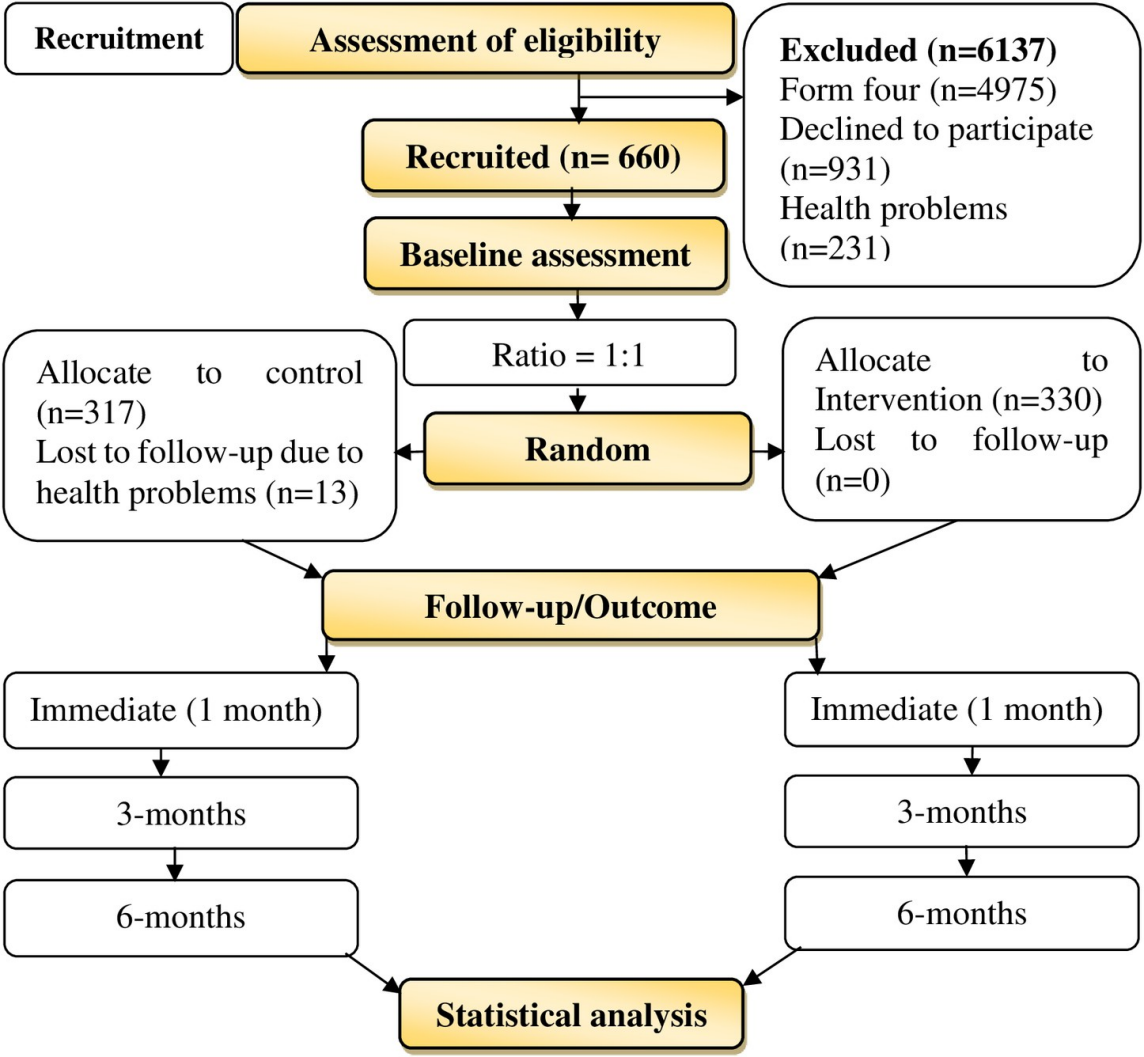
Flow diagram showing the recruitment of adolescents, their distribution by research arms, and study timelines.

A voluntary basis was used as a criterion for the participants to join the study. Thus, adolescents were free to withdraw from the study at any point without being questioned but their information would not be processed and analysed. Written informed consent was obtained from all participants who were above 18 years and teachers and or legally acceptable representatives consented in writing for minors (below 18 years of age) as one of the criteria to participate in the study. The principal investigator and research assistants gave adolescents and or parents a brief description of the aim and merits of participating in the study before they would opt to join. Recruitment of adolescents was conducted once before the administration of baseline assessment and commencement of the intervention by the researcher independent of this study. The inclusion criteria such as being at ordinary level secondary school, age between 12 and 19 years, exposed to SRH lesson materials, and being a free individual from other projects were used to assess adolescents who were eligible to join the study. As shown in Fig 1, the study excluded form four adolescents who were preparing themselves for their final form four national examinations.

### Procedure of randomization

A randomization procedure was performed immediately after baseline assessment by researchers not part of the study. This followed after brief descriptions given to adolescents about it alongside their confirmation to be ready to be randomized. Three researchers not part of this study who were assigned names of the schools performed randomization of schools. All the public ordinary-level secondary schools in the sampled districts (n = 4) were considered eligible for the study. However, they were randomly selected using a simple random sampling technique (by lottery method) to get 3 schools from each district to reflect the study arms (n = 12 secondary schools). Via the use of three random allocation cards (each card was written the name of the intervention such as pure PBP, hybrid PBP, and LBP), the independent researchers then, drew one out of three cards to identify the school arms.

The randomization pattern ensured that adolescents in different groups received different interventions and did not exchange information. The necessary sample of the study was determined by using G*Power computer software program version 3.1.9.7 as recommended to be used in calculating the desired sample in randomized controlled trials by previous studies [35]. On one side, the F-tests set at a statistical test of linear multiple regression with fixed model and R2 deviation from 0 of prior power of analysis was used to determine the minimum sample size set at α err probability = 0.05, power (1 – β err probability) = 0.80, and small effect size (Cohen’s d) = 0.02 (a statistical power of the study).

A total of 660 (97.1%) out of 6797 adolescents were recruited to participate in the study. However, 6137 adolescents were excluded due to different reasons. Among them, 4975 (81.1%) were excluded because they were form four adolescents who were in preparations for their final form four examination, 931 (15.2%) adolescents declined to join the study, and 231 (3.7%) were adolescents due to various health problems. The study adopted the set at 0.05 and statistical power of 80% set at 1.0 to randomly allocate adolescents into research arms by a ratio of 1:1 (control group: n= 330 and intervention: n= 330). However, thirteen adolescents in the LBP group (control) lost follow-up after the baseline assessment.

A cluster-based approach was used to allocate adolescents to participate in the study in either the pure PBP, hybrid PBP, or LBP groups by the researcher independent of the study. The random allocation schemes were generated via three simple cluster randomization procedures (random number table). A random numbers table sampling method was employed to achieve a proportion of adolescents per class. Every adolescent stayed in his or her assigned intervention arm for the duration of the study. Criteria such as adolescents’ age were used to randomize them into the research arms.

### Blinding procedure

Through the principal investigator, the study employed a double-blinding design by not letting the research assistants and adolescents know whether they were in a control or intervention group. This was done to make them comply with the intervention regimes and minimize the adjustment of an intervention. Moreover, the outcome assessor and data analyst were also blinded to the study allocations.

### Data collection instruments

The study collected primary data using 15 items pre-tested Sexual-risk behaviour Beliefs and Self-esteem Scale (SRBBSES). Tight, Mok, and Huisman [36] and Unis *et al*., [37], recommended the adopted instrument when researchers intend to assess skills for sexual behaviour among young people. This study measured soft skills based on self-reported intentions to demonstrate safe sexual behaviours among adolescents.

### Validity and reliability of the research tools

The questionnaires were anonymously filled in after being translated from English into the Swahili language to facilitate understanding of the items among adolescents. To ensure confidentiality, adolescents’ names were not included in the questionnaires. The inter-rater design was employed to assure content validity whereas 91 questionnaire items were distributed among five experts on SRH to ascertain whether the content of questionnaires was appropriate among adolescents and to the purpose of the study. Each expert rated the relevance of each questionnaire item using a 4-point Likert scale (0 = not relevant, 1 – somewhat relevant, 2 = relevant, and 4 = very relevant). The level of item retention was set at >0.8 to establish the content validity index. Thus, any item not meeting the specified requirement was dropped for further required modifications [38–40].

The modified questionnaires were then administered among 10 randomly selected adolescents who were already exposed to SRH education from the school that was sampled for piloting research tools to complete the face validity form developed by the principal investigator. The form consisted of questionnaire items on a 4-point Likert scale ranging from 1 = strongly disagree to 4 = strongly agree. The items were distributed among adolescents in a classroom setting. The evaluation form aimed to determine issues around usability, readability, consistency of the layout, style, and formatting, and the clarity of the language used in the questionnaires.

The consensus view was that the research tools provided a suitable means of assessing the variables under study, no item was disagreed by adolescents and most of them agreed on the readability of the items (93.2%), consistency of the item layout (98.8%) and clarity of wordings (language) used (96.1%). Two (2/10) out of ten responses showed disagreement. Adolescents’ responses confirmed that the tools demonstrated face validity at ≥95%. The questionnaires were then pretested among 240 adolescents to determine a Cronbach’s Alpha for their internal consistency reliability. Exploratory factor analysis was first performed for data reduction to get high weighed items above the suggested statistical thresholds (>0.3) as recommended by authors from previous studies [38–40].

The correlation coefficient was set at a cut-off point of ≥0.30 whereas, the Kaiser-Meyer-Oklin (KMO) value of ≥0.05 and p<0.05 was used to assess sampling adequacy and was set at a cut-off point of ≥0.60. Moreover, Bartlett’s test of sphericity was used to support the factorability of the correlation matrix and examine whether the original data were appropriate for factor analysis. The suitability of data for factor analysis was assessed whereas, an inspection of the correlation matrix revealed the presence of coefficients of ≤0.30. The KMO was 0.603, which was approximately equivalent to the cut-off point of 0.6; p<0.05, and Bartlett’s test of sphericity was statistically significant [(χ^2^ = 5281.036 (2278); p<0.01)]. A Cronbach Alpha was significantly above the recommended measurements (0.949; M=17.85±14.851; variance=220.555).

### Primary predictors

The independent variables included the interventions (integrated RH lesson materials in a PBP) and sociodemographic characteristics of adolescents such as age, sex, religion, class level, and parental characteristics whose effects on sexual behaviour among adolescents were determined at the end of the study via analytical procedures. As proposed by previous studies [41] sex was used instead of gender to explain adolescents’ biological influence over the primary of interest.

### Primary outcomes

The primary outcome was a soft skill that considered self-reported intentions to demonstrate safe sexual behaviours. The variable was measured by using four-point Likert scale responses ranging from “0 points” for not being sure to perform the behaviour asked in the item and the “4 points” for being extremely sure to perform a particular behaviour. The tool was divided into sub-categories including adolescents’ ability to abstain from sexual behaviour (24-items); self-reported intention to use a condom (4-items); ability to withstand sexual dilemma (16-items) and adolescents’ ability to withstand sexual coercion (60-items). The Likert scale responses were then transformed to compute a new dichotomized variable that represented adolescents’ levels of soft skills. The maximum possible scores for soft skills for safe sexual behaviour were computed as a sum of each item scores ranging from 0 scores (poor soft skills) to higher scores (good soft skills). There were no changes in the trial outcomes after the commencement of the study.

### Data analysis

The study adopted randomized-groups and repeated-measures analysis approach to analyse quantitative data via SAS computer program version 9.4. The main analytical strategy to minimize bias, missing data, and inequality of information that would occur during the allocation of adolescents to the intervention groups and loss of follow-ups, the intention-to-treat analysis (ITT) was opted for. To detect 50% differences in sexual behaviour among adolescents, (α = 0.05, β = 0.80, σ = 2.8) statistical values were used. Inferential statistics were determined through Difference-in-difference (D-I-D) analysis that is used in the study of longitudinal cohort data with pre-and post-exposure repeated measures. The D-I-D analysis approach is typically used to estimate the effect of specific interventions by comparing changes in outcomes over time between the population involved in the intervention and the population that is not (the control group). It allows the comparison of changes over time in the outcome between exposed and control treatment arms while accounting for controlling the possible confounding variables [42].

The D-I-D design measures the change in the outcome between two-time points (the pre-and post-periods) for an exposed treatment arm and a control treatment arm, then subtract one from the other to see the difference in the differences between the treatment arms. To perform a D-I-D analysis, the control group was treated independently by estimating the mean score difference between pre-test and post-test (within-group difference) to get the extent of change of adolescents’ soft skills for the safe sexual behaviour of that particular group. The same procedure was performed in the intervention group to establish the extent of change of adolescents’ mean scores of soft skills for safe sexual behaviour between pre-test and post-test. Thereafter, a change obtained in the intervention group minus the change obtained in the control group brings a discriminated effect of an intervention.

In this study, a difference-in-difference analysis was performed using Linear Mixed Model (LMM) to assess the effect of the intervention on soft skills for safe sexual behaviour, and sexual behaviour among adolescents. For soft skills, the post-period (end-line) measurements were taken after one month from a baseline while its retention post-period measured was considered after three and six months. The intervention consisted of three treatment arms: Lecture Group (Control), hybrid PBP, and pure PBP. The model accounted for the repeated measures of the outcome variables.

The general fixed effect of D-I-D LMM can be presented using the following equation:

$$\log it(\pi (x))=\ln(\frac{\pi (x)}{1-\pi (x)})=\beta_{0}+\beta_{1}*Time+\beta_{2}*Treatment+\beta_{3}*Time*Treatment$$ (1)

Whereas the chance of having outcome for subjects with characteristics x at time t, Time is a dummy variable for a period, equal to 1 when the outcome measurement was made in the post-period (end-line) and 0 for baseline measurements. Treatment is a dummy variable for subject treatment arm membership. The composite variable Time* Treatment is the interaction between time and treatment. In the model above, the parameter β_0_ represents the intercept, the log odds of having an event of interest in the control treatment arm at the baseline measurement. The β_1_ is the change in log odds (log odds ratio) of having an event of interest in the control treatment arm between baseline and end-line.

The parameter β_2_ indicates the difference in log odds of having an event of interest between the treatment and control treatment arm at baseline, whereas the coefficient β_3_ (interaction) measures the difference in slopes between the two treatment arms (hybrid PBP versus control or pure PBP versus control). The coefficient of the interaction term provides the estimate and inference of the difference-in-differences between the two treatment arms.

In the model results, if this coefficient estimate is statistically significant, it indicates that the slopes in the two treatment arms are not parallel. Therefore, the intervention has affected the outcome in the treatment arm differently unlike the underlying background trend, as taken by the control treatment arm. The D-I-D LMM presented above does not mean that it differs significantly from a more conventional LMM approach to regression. It is just a specification used in this study.

### Study timelines

**Fig 2:**
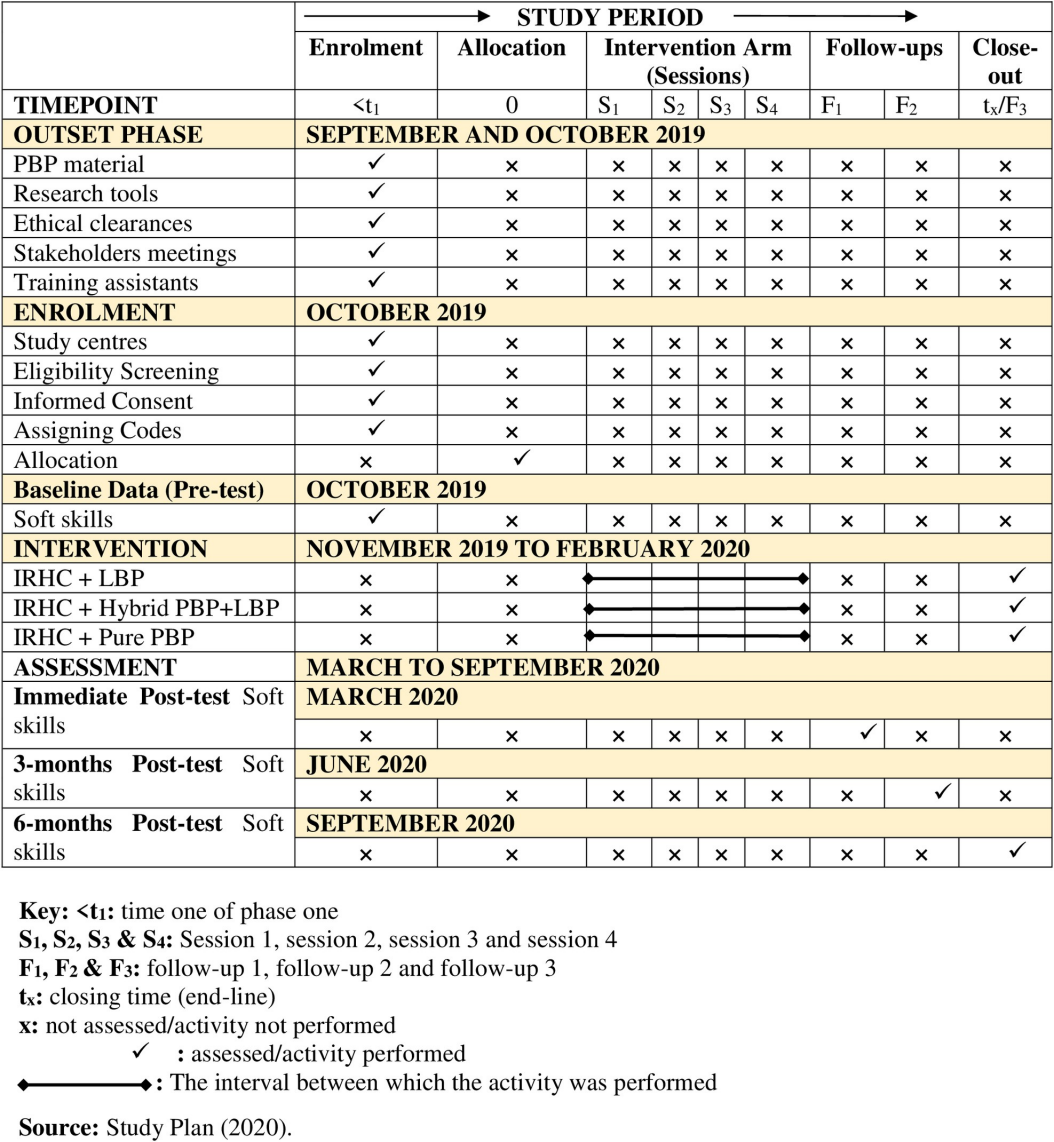
The study timelines alongside study activities.

As shown in Fig 2, the study was conducted using a longitudinal design incorporating a multicentre approach from September 2019 to September 2020 in five consecutive phases to ascertain the effect of the intervention on adolescents’ sexual behaviour over time. The end date of the study was 12 months after the baseline intervention. First, the outset phase consisted of 2 months (September to October 2019) for ethical approval, permits, and clearances. Thereafter, the development of the integrated RH lesson materials in a PBP, evaluation of research instruments, stakeholder meetings, recruitment, and training of research assistants about the tools, data collection, and implementation of the interventions, and pre-testing of research tools was performed.

There followed the enrolment of the eligible study locations and consented adolescents in October 2019. A baseline survey (Baseline data collection) and preparation of the outset report were then performed in mid-October 2019 among the sampled secondary schools. The second phase focused on the implementation of the intervention from November 2019 to February 2020 (for four months). This phase included the actual delivery of SRH lesson materials between groups by using pure PBP, hybrid PBP, and LBP respectively. The third phase consisted of follow-up assessments of sexual behaviours among adolescents from March to September 2020. A 3-month post-intervention assessment, which was administered in June 2020, and a 6-months post-intervention assessment that was administered in September 2020, were performed respectively. Each phase involved data collection, data processing, and management, analysis, and report writing.

## Results

**Fig 3:**
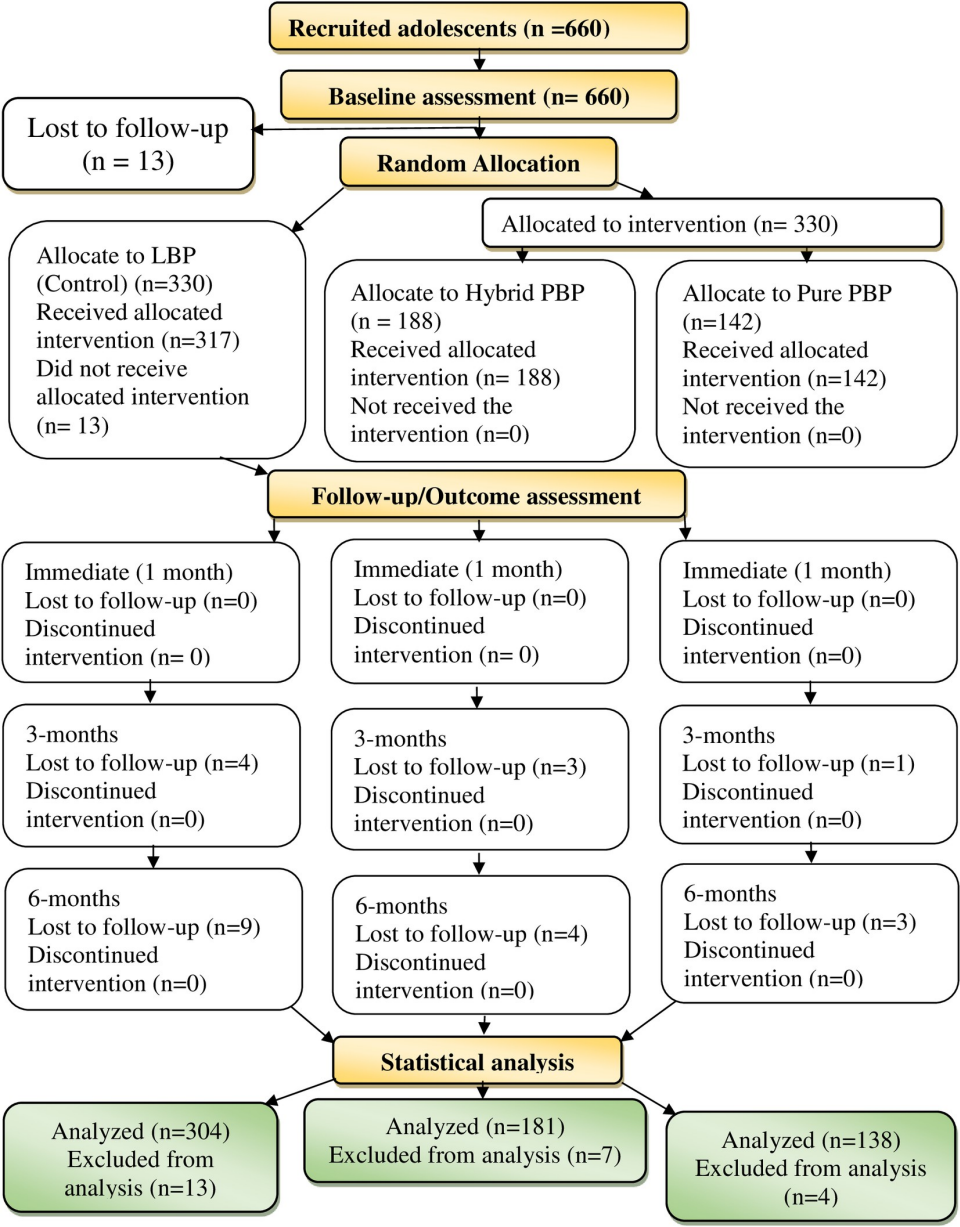
Flow diagram indicating adolescents’ adherence to the interventions by research arms throughout the study timelines.

Findings in Fig 3 indicates the enrolled adolescents alongside frequencies of adherence and loss of follow-ups among them throughout study timelines.

### Adolescents’ sociodemographic characteristics profiles by Study Arms

Baseline findings in Table 2 show that adolescents did not differ significantly in some sociodemographic characteristics profiles between groups (p>0.05). However, they differed in religion, class, disabilities, fathers’ education, with whom were they living, head of the household, and sexual ideology (p<0.05), the factors, which were then controlled during analysis to discriminate the effect of the intervention. The mean age of the adolescents was 15±1.869 years (minimum = 12 years; maximum = 19 years). The most dominant age group (71.2%) ranged between 13 to 16 years whereas, 72.5% (n = 103) of adolescents were found in pure PBP, 66.5% (n = 125) in hybrid PBP and 73.5% (n = 233) in a LBP group. The proportion of female adolescents was 57.5% between groups compared to males. A 58.5% (n = 83) of female adolescents were found in a pure PBP, 58.5% (n = 110) in hybrid PBP and 56.5% (n = 179) in a LBP group. A 58.3% (n = 377) of adolescents were sexually active by engaging in at least one unsafe sexual behaviour including sexual relationship (39.9%), sexual intercourse (5.7%) multiple sexual partners (8.7%), and not using a condom during sexual intercourse (96.0%).

**Table 2:** Proportions of Adolescents’ sociodemographic characteristics profiles by study arms (n = 660).

**Variables**	**LBP Group N = 317**	**Hybrid PBP N = 188**	**Pure PBP N = 142**	**Chi-square (p-value)**
	**n (%)**	**n (%)**	**n (%)**	
**Age (years)**				
Mean = 15±1.869				
Min = 12				
Max = 19				
**Age group (years)**				5.54(0.2367)
10-12	31(9.39)	18(9.57)	9(6.34)	
13-16	243(73.64)	125(66.49)	103(72.54)	
17-19	56(16.97)	45(23.94)	30(21.13)	
**Birth order**				1.64(0.4415)
First born	208(63.03)	128(68.79)	92(64.79)	
Last born	122(36.97)	60(31.91)	50(35.21)	
**Sex**				0.27(0.8739)
Female	169(51.21)	110(58.51)	83(58.45)	
Male	161(48.79)	78(41.49)	59(41.55)	
**Religion**				**17.75(0.0001)**
Christian	123(37.27)	41(21.81)	34(23.94)	
Muslim	207(62.73)	147(78.19)	108(76.06)	
**Orphan**				0.61(0.7374)
No	283(85.76)	168(89.36)	130(91.55)	
Yes	47(12.24)	20(10.64)	12(8.45)	
**Class**				**25.70(0.0001)**
Form I	173(52.42)	71(37.77)	44(30.99)	
Form II	62(18.79)	56(29.79)	56(39.44)	
Form III	95(28.79)	61(32.45)	42(29.58)	
**Any disability**				**8.20(0.0165)**
No	319(96.67)	177(94.15)	130(91.55)	
Yes	11(3.33)	11(5.85)	12(8.45)	
**Parent disability**				0.34(0.8446)
No	318(96.36)	181(96.28)	137(96.48)	
Yes	12(3.64)	7(3.72)	5(3.52)	
**Father education**				**14.08(0.0287)**
No formal education	55(16.67)	48(25.53)	45(14.2%)	
Primary	137(41.52)	64(34.04)	134(42.3%)	
Secondary	83(25.15)	51(27.13)	83(26.2%)	
College/university	55(16.66)	25(13.30)	55(17.4%)	
**Mother education**				4.11(0.3125)
No formal education.	91(27.58%)	50(26.6%)	84(26.5%)	
Primary	179(54.24%)	87(46.3%)	150(47.3%)	
Secondary	37(11.21%)	13(6.9%)	19(6.0%)	
Collage/university	23(6.97%)	38(20.2%)	64(20.2%)	
**Occupation of Father**				0.37(0.8453)
Self Employed	265(80.31%)	157(83.5%)	129(90.8%)	
Employed	47(14.24%)	22(11.7%)	7(4.9%)	
Not working	18(5.45%)	9(4.8%)	6(4.2%)	
**Occupation of Mother**				0.19(0.9437)
Self Employed	285(86.36%)	154(81.9%)	120(84.5%)	
Employed	23(6.97%)	14(7.4%)	2(1.4%)	
Not working	22(6.67%)	20(10.6%)	20(14.1%)	
**Living with**				**15.53(0.0165)**
Both Parents	214(64.85)	94(50.0)	92(64.79)	
Father only	17(5.15)	11(5.85)	9(6.34)	
Mother only	47(14.24)	29(15.43)	18(12.68)	
Relatives	52(15.76)	54(28.72)	23(16.20)	
**Family type**				4.21(0.1216)
Nuclear Family	188(56.97)	92(48.94)	67(47.18)	
Extended Family	142(43.03)	96(51.06)	75(52.82)	
**Household head**				**22.03(0.0002)**
Father	249(75.45)	132(70.21)	129(90.85)	
Mother	45(13.64)	26(13.83)	5(3.52)	
Relative	36(10.91)	30(15.96)	8(5.63)	
**Parent-adolescent SRH communication**				0.16(0.9240)
No	243(73.64)	139(73.94)	105(73.94)	
Yes	87(26.36)	49(26.06)	37(26.06)	
**Travelled in last 12 months**				3.16(0.2055)
No	133(40.30)	64(34.04)	49(34.51)	
Yes	197(59.70)	124(65.96)	93(65.49)	
**Parent Financial protection**				4.84(0.0887)
Yes	118(35.76)	59(31.38)	37(26.06)	
No	212(64.24)	129(68.62)	105(73.94)	
**Social cohesion**				0.05(0.9767)
Yes	243(73.64)	139(73.94)	105(73.94)	
No	87(26.36)	49(26.06)	37(26.06)	
**Sexual Ideology**				**13.49(0.0012)**
Negative	226(68.48)	135(71.81)	119(83.80)	
Positive	104(31.52)	53(28.19)	23(16.20)	
**Media Exposure**				0.63(0.7313)
Yes	322(97.58)	185(94.40)	141(99.30)	
No	8(2.42)	3(1.60)	1(0.70)	
**Drugs Exposure**				1.24(0.5378)
Yes	48(14.55)	23(12.23)	15(10.56)	
No	282(85.45)	165(87.77)	127(89.44)	
**Sexual behaviour**				2.331(0.1864)
Never	147(46.37)	77(40.96)	46(32.39)	
Sexual relationship	107(33.75)	85(45.21))	66(46.48)	
Sexual intercourse	17(5.36)	11(5.85)	9(6.35)	
Multiple sexual partners	34(10.73)	9(4.79)	13(9.15)	
Condom use	12(3.79)	6(3.19)	8(5.63)	

**Source:** Filed Data (2020).

### Proportions of soft skills for safe sexual behaviours by sociodemographic characteristic profiles among adolescents between baseline and end-line assessments

A descriptive statistical analysis was performed to determine the frequencies and proportions of soft skills by sociodemographic characteristic profiles among adolescents. The findings in Table 3 show that 73.6% (n = 231) of the adolescents with improved intent to practise safe sexual behaviours were in the middle adolescence stage (13 to 16 years). Females (55.4%), Form I (40.1%), and able-bodied (93.9%) adolescents had more improved soft skills for safe sexual behaviours than their counterparts males, form II & III, and disabled adolescents respectively. Adolescents who were living in nuclear families (50.3%), or living with both parents (60.8%) demonstrated improved soft skills compared to others. Moreover, adolescents who had never communicated with their parents about SRH matters (75.8%), those with parental financial protection (70.4%), had opportunities to join social groups (74.1%), had positive sexual belief (74.5%), were exposed to media (99.0%), and did not engage in drug abuse (86.9%) after the commencement of the study demonstrated a significant intent to practise safe sexual behaviours than their counterparts respectively. The findings of other sociodemographic characteristic profiles of adolescents against proportions of soft skills are shown in Table 3.

**Table 3:** Proportions of the overall soft skills by sociodemographic characteristic profiles among adolescents between baseline (12-months before the study) and end-line assessments (6-months of the study).

Variable	Baseline(N = 92/660)	End-line (N = 314/647)
	Soft skills (Yes) n (%)	Soft skills (Yes) n (%)
**Districts**		
The City Council of Dodoma	36(39.1%)	101(32.2%)
Kondoa District Council	24(26.1%)	100(31.8%)
Lindi Municipal Council	17(18.5%)	55(17.5%)
Kilwa District Council	15(16.3%)	58(18.5%)
**Age Groups**		
10 to 12 yrs.	6(6.5%)	19(6.0%)
13 to 16 yrs.	66(71.8%)	231(73.6%)
17 to 19 yrs.	20(21.7%)	64(20.4%)
**Birth space**		
1st Born	56(60.9%)	201(48.1%)
Last Born	36(39.1%)	113(49.3%)
**Gender**		
Male	29(31.5%)	140(44.6%)
Female	63(68.5%)	174(55.4%)
**Religion**		
Christian	24(26.1%)	87(27.7%)
Muslim	68(73.9%)	227(72.3%)
**Orphanage**		
Yes	8(8.7%)	28(8.9%)
No	84(91.3%)	286(91.1%)
**Year of study**		
Form I	43(46.7%)	126(40.1%)
Form II	17(18.5%)	95(30.3%)
Form III	32(34.8%)	93(29.6%)
**Any Disability**		
Yes	7(7.6%)	19(6.1%)
No	85(92.4%)	295(93.9%)
**Parents’ Disability**		
Yes	6(6.5%)	13(4.1%)
No	86(93.5%)	301(95.9%)
**Father’s education**		
Never gone to School	21(22.8%)	65(20.7%)
Primary Education	39(42.4%)	122(38.9%)
Secondary Education	17(18.5%)	81(25.8%)
College/University	15(16.3%)	46(14.6%)
**Mother’s education**		
Never gone to School	26(28.3%)	82(26.1%)
Primary Education	49(53.3%)	151(48.1%)
Secondary Education	4(4.3%)	23(7.3%)
College/University	13(14.1%)	58(18.5%)
**Father’s Occupation**		
Self Employed	76(82.6%)	263(83.8%)
Employed	10(10.9%)	33(10.5%)
Not working	6(6.5%)	18(5.7%)
**Mother’s Occupation**		
Self Employed	75(81.5%)	260(82.8%)
Employed	5(5.4%)	18(5.7%)
Not working	12(13.1%)	36(11.5%)
**Living With**		
Both Parents	62(67.4%)	191(60.8%)
Father only	3(3.2%)	15(4.8%)
Mother only	11(12.0%)	43(13.7%)
Relative/Friends	16(17.4%)	65(20.7%)
**Type of Family**		
Nuclear	43(46.7%)	158(50.3%)
Extended	49(53.3%)	156(49.7%)
**Head of Household**		
Father	70(76.1%)	241(76.8%)
Mother	10(10.9%)	32(10.2%)
Relative	12(13.0%)	41(13.0%)
**Communicated SRH matters with parents**		
Yes	20(21.7%)	76(24.2%)
No	72(78.3%)	238(75.8%)
**Travel away for more than a month**		
Yes	59(64.1%)	209(66.6%)
No	33(35.9%)	105(33.4%)
**Parental Financial Protection**		
No	21(22.8%)	93(29.6%)
Yes	71(77.2%)	221(70.4%)
**Social Cohesion**		
Yes	69(75.0%)	233(74.2%)
No	23(25.0%)	81(25.8%)
**Sexual belief**		
-ve	21(22.8%)	80(25.5%)
+ve	71(77.2%)	234(74.5%)
**Exposure to Media**		
Yes	91(98.9%)	311(99.0%)
No	1(1.1%)	3(1.0%)
**Drug Abuse**		
Yes	16(17.4%)	41(13.1%)
No	76(82.6%)	273(86.9%)

**Source:** Filed Data (2020).

### The proportions of adolescents’ soft skills for safe sexual behaviours between groups across the study timelines

**Fig 4:**
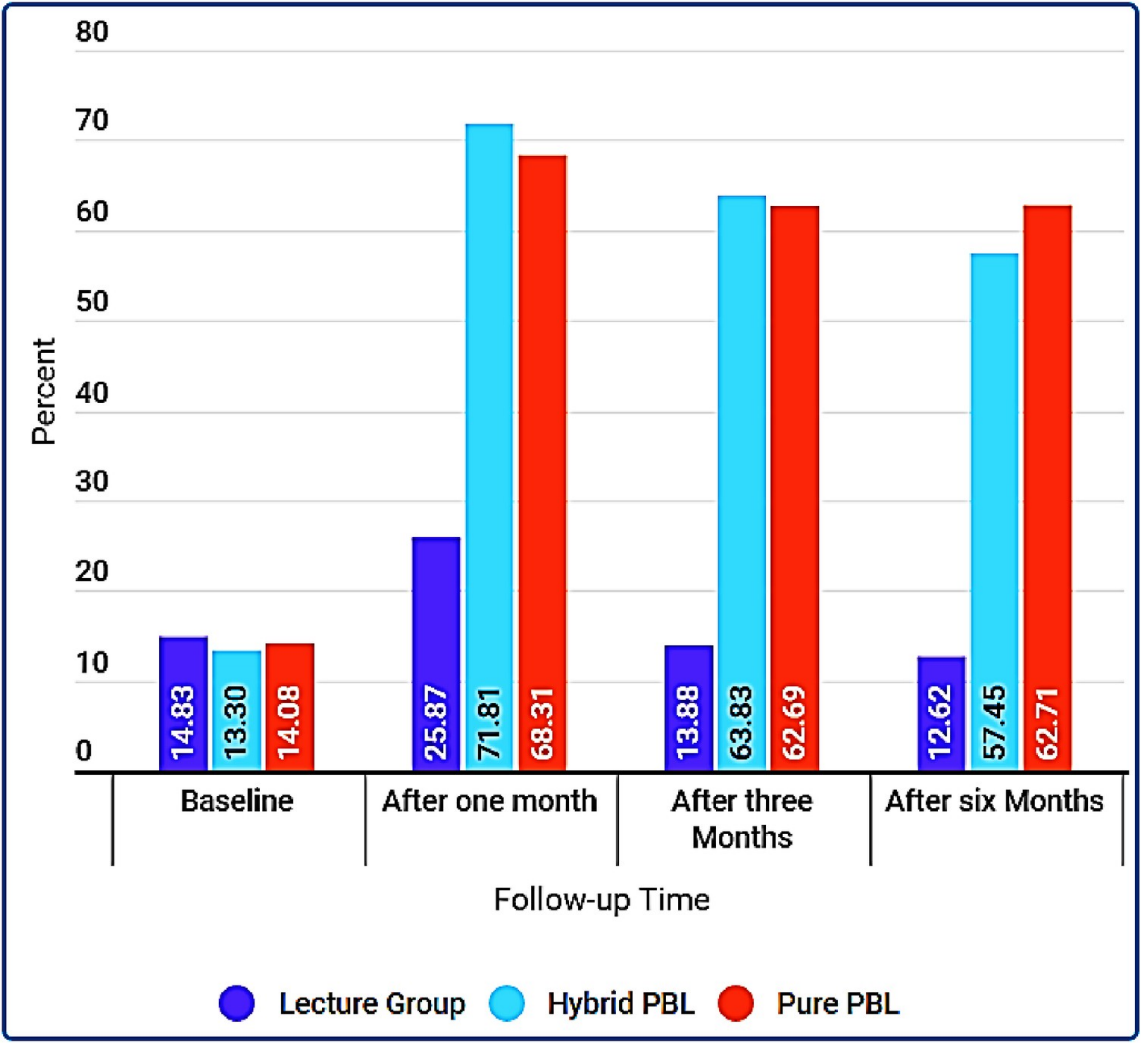
Proportions of adolescents’ soft skills for safe sexual behavior between groups across the study timelines.

Findings in Fig 4 indicate that the proportions of adolescents’ intent to practise safe sexual behaviours was significantly similar at baseline (pure PBP = 14.08%, hybrid PBP = 13.30% and LBP = 14.83%; p>0.05). However, there was a significant difference immediately after the interventions (p<0.05). Adolescents in the hybrid PBP intervention (71.81%) demonstrated improved soft skills for safe sexual behaviour followed by adolescents in the pure PBP intervention (68.31%) against their counterparts in the LBP group (25.87%). Although their skills showed to decrease over time, the findings of a 3-month follow-up showed that a significant proportion of adolescents in the hybrid PBP (63.83%) and the pure PBP (62.69%) demonstrated a significant retention rate of soft skills for safe sexual behaviours than adolescents in the LBP group (13.88%). Moreover, adolescents in the pure PBP (62.71%) and hybrid PBP (57.45%) had more improved retention rate of soft skills than adolescents in the LBP group (12.62%) when assessed at 6-months follow-up. Adolescents in the pure PBP had a higher retention rate of soft skills at 3-months follow-up than at 6-month follow-up compared to adolescents in the hybrid PBP and LBP groups respectively.

### The effect of integrated RH lesson materials in a PBP on soft skills for safe sexual behaviours among adolescents

The study determined the empirical-based parameter estimates (β) of the adjusted LMM for the difference in difference analysis of soft skills for safe sexual behaviour among adolescents. The findings in Table 4 showed that, at baseline, no significant difference was found in soft skills about SRH for subjects in hybrid PBP (β=0.1949, p=0.7328; 95%CI: 0.0681, 1.0073), and pure PBP (β =0.5091, p= 0.4236; 95%CI: 0.0198, 1.0871) in comparison to those in the control group. This implies that the soft skills score for safe sexual behaviour among subjects in the three treatment groups was not different at baseline. The mean soft skill scores on safe sexual were observed to be higher at the end-line compared to baseline for all three treatment arms. The magnitude of the difference calculated from the fitted model showed that the estimated mean soft skills score among adolescents increased significantly at post-test (β=3.8801; p<0.01; 95%CI: 1.0087, 5.1290) than at baseline.

**Table 4:** The effect of integrated RH lesson material in a PBP on soft skills for safe sexual behaviours among adolescents.

Effect	Estimate (β)	Std. Error	95%CI		p-value
			Low	Up	
Intercept	44.9546	0.6372	31.0330	47.9801	0.0001
Time					
After 1 Month	3.8801	0.4372	1.0087	5.1290	0.0001
After 3 Months	2.0044	0.1336	1.0234	4.1182	0.0001
After 6 Months	1.9803	0.1178	0.8399	3.1099	0.0001
Baseline	Reference				
**Treatment**					
Hybrid PBP	0.1949	0.5706	0.0681	1.0073	0.7328
Pure PBP	0.5091	0.6358	0.0198	1.0871	0.4236
LBP Group	Reference				
**Religion**					
Christian	-0.1289	0.4202	-0.0769	0.9167	0.7591
Muslim	Reference				
**Class**					
Form I	Reference				
Form II	-0.4068	0.4682	-0.0844	0.8934	0.3852
Form III	-0.1272	0.4430	-0.0982	0.2103	0.7742
**Disability**					
No	Reference				
Yes	-0.2370	0.8709	-0.0485	0.18621	0.7856
**Father education level**					
No formal education	Reference				
Primary	-0.4560	0.5319	-0.0532	0.1382	0.3916
Secondary	-1.0310	0.5670	-0.8993	-3.0943	0.0695
Collage/university	-0.7397	0.6619	-.07194	-1.3331	0.2642
**Living with**					
Both Parents	Reference				
Father only	-2.0037	0.8198	-1.0291	-4.0345	0.0148
Mother only	-0.4917	0.6265	-0.0123	-8.1124	0.4328
Relatives	-0.7405	0.5887	-0.1328	-1.9234	0.2090
**Household head**					
Father	Reference				
Mother	0.3805	0.6720	0.1228	0.5025	0.5715
Relative	1.0912	0.4209	0.8118	2.9441	0.1274
**Sexual Ideology**					
Negative	Reference				
Positive	-0.5157	0.1927	-0.0341	-0.9220	0.2209
	**Estimate (β)**	**Std. Error**	**95%CI**		**p-value**
Time*Treatment			**Low**	**Up**	
Time* Hybrid PBP	9.0986	0.7166	4.7772	14.2311	0.0001
Time* Pure PBP	8.7114	0.7861	3.9990	10.1208	0.0001
**D-I-D Coefficient**					
**Label**	**Estimate (β)**	**Std. Error**	**95%CI**		**p-value**
			**Low**	**Up**	
Hybrid PBP vs LBP group	9.0986	0.7166	4.7772	14.2311	0.0001
Pure PBP vs LBP Group	8.7114	0.7861	3.9990	10.1208	0.0001
Hybrid PBP vs Pure PBP	0.3872	0.8656	0.1098	0.6321	0.6548

**Source:** Field data (2020).

Despite the decrease in the retention rate of soft skills scores among adolescents over time, findings in Table 4 indicate that the scores were significantly higher at 3-months follow-up (β=2.0044; p<0.01; 95%CI: 1.0234, 4.1182) and at 6-months follow-up (β=1.9803; p<0.01; 95%CI: 0.8399, 3.1099) than at baseline assessment. The magnitude of the adjusted D-I-D regression coefficient by the time for the hybrid PBP was 9.0986, and it was significant at α=0.05 (p<0.0001; 95%CI: 4.7772, 14.2311). This means that the change in soft skills score for safe sexual behaviour from baseline to end-line was significantly higher among adolescents in the hybrid PBP group compared to adolescents in control. Equally, there was a significantly higher improvement in soft skills for safe sexual behaviour when moving from the baseline to the end line among adolescents in the pure PBP group compared to those in control (β=8.7114, p<0.0001; 95%CI: 3.9990, 10.1208). Nevertheless, no significant difference in change in knowledge score was observed for respondents in hybrid PBP and pure PBP treatment arms (β=0.3872, p=0.6548; 95%CI: 0.1098, 0.6321). Thus the school-based training intervention had a significant positive influence on improving the intent to practise safe sexual behaviour among secondary school adolescents in Tanzania.

## Discussion

Contrary to baseline findings, post-test results revealed that adolescents exposed to integrated RH lesson materials in a PBP can demonstrate soft skills for safe sexual behaviours characterized by self-reported intentions to abstain from sexual relationships and sexual intercourse, having informed decisions to negotiate on the use of a condom, withstand sexual emotions, coercions, and or sexual dilemmas. The findings imply that RH lesson materials can be integrated and implemented in a PBP for it to demonstrate a substantial effect in enhancing soft skills for safe sexual behaviour among adolescents in ordinary level secondary schools in Tanzania.

The post-test findings indicated that adolescents were able to demonstrate intentions to say “NO” firmly to anyone who would want to have sexual relationships and sexual intercourse with any of them and stick to it in their day-to-day lives. Moreover, adolescents reported intentions to find a friend who also would wish not to have a sexual relationship and or sexual intercourse until they married each other later in their life. Yet, they reported their intentions to abstain from sex or else use a condom and readiness to withstand sexual dilemmas. Additionally, significant number of adolescents demonstrated intentions to say “NO” or report to the appropriate authority if families, relatives, or friends forced them to marry during their young ages.

Some adolescents reported intentions to say “NO” to sexual relationships or intercourse if someone would ask them to do so in exchange for some money, material gain, or as a business. Others appeared to be anxious about saying “NO” to any form of forced sexual relationship and or sexual intercourses be it by peer friends, elder people, or any strange individuals, and not keeping secrets if someone would touch or force them to watch sexual pictures, videos, or touch someone’s private parts. The study acknowledges that its findings were limited to no studies that demonstrated the effect of PBP on enhancing soft skills for safe sexual behaviours among adolescents in ordinary level secondary schools in Tanzania. However, the findings outlined in this study tally with those found in some previous experimental and non-experimental studies that tested the PBP in other variables.

The meta-analysis by Zhou *et al*., [42] indicated that the implementation of problem-based pedagogy was significantly associated with not only improved independent analysis skills but also students’ learning habits, team spirit, and or oral expression. A match in findings on the effect of PBP on psychological outcomes between these two studies may imply that a pedagogy holds a significant effect in developing adolescents who are critical thinkers and problem solvers. Evidence about the effect of PBP on soft skills among learners has been demonstrated by Deep *et al*., [43] in Malaysia. Although they studied undergraduate students, their findings showed that the adoption and implementation of problem-based pedagogy can enhance the development of learners with soft skills alongside improving group learning, including overcoming communication conflicts, which may occur among them.

Despite differences in study locations, research methodology, and study population, the findings on the effect of PBP on soft skills tally with the findings of this study. It appears that the implementation of PBP as an interactive learning strategy can enhance soft skills among adolescents to make reasoned and informed decisions over their sexual behaviours. Although Idrus *et al*., [44] examined the effect of problem-based pedagogy on soft skills among engineering students, the findings revealed that 77.7% of the PBP facilitators demonstrated preferences of implementing the pedagogy in their engineering courses.

Moreover, their findings revealed that with the implementation of PBP, students would be developed with good critical thinking, communication, teamwork, and problem-solving skills. Despite the differences in study variables, the findings of the two studies tally on the aspect of the effect of PBP in influencing change among adolescents whereby a pedagogy has demonstrated a significant effect in shaping adolescents’ behavioural outcomes. All the same, Demirel [45], through a meta-analysis, determined the effect of PBP on students’ attitudes to learning in Turkey. The study found that the PBP helped students to gain a positive attitude towards the courses they were learning. Students demonstrated a sense of independence from teachers, continued to learn along with their lives, and gained confidence and a positive attitude to their being.

Thus, PBP was well thought out to influence participatory and collaborative learning among students to the level that it could make them be responsible for their learning activities with minimal support from teachers. The findings of the current study, tally with the findings observed by Unis *et al*., [37] who assessed rural high students’ sexual behaviour and self-esteem at the age of 16 to 18 years in Sweden. They found that students had both high basic and earned self-esteem about safe sexual behaviours whereby most of them reported having few sexual partners. Their findings implied that the more students advanced in age in good innovative and nurturing pedagogies facilitating SRH learning, the more they developed self-esteem over unsafe sexual behaviours.

Additionally, the findings observed by Tadjer *et al*., [46] demonstrated that students’ soft skills and cognitive skills in parallel, become significantly improved when they are developed under a problem-based learning environment than other pedagogies can do. Students become critical thinkers and problem-solvers by undergoing experimental processes and inquiry learning when they are exposed to real-world or ill-structured hypothetical problems. Differences in study locations, research methodology, and study population do not exempt the tallying of their findings with this study as they both examined the effect of PBP on soft skills among participants. The findings may imply that the implementation of PBP can enhance soft skills for safe sexual behaviours among adolescents.

Generally, this study provides a piece of research-based evidence that the integrated RH lesson materials in a PBP (be it pure or hybrid) may not merely influence adolescents’ intent to practise safe sexual behaviours but also can serve as a formal guide to teachers and or health workers in facilitating SRH learning to adolescents in ordinary-level secondary schools in Tanzania.

## Conclusion

Adolescents’ intent to say “NO” to sexual relationships or sexual intercourse and stick to it, avoid multiple sexual partners, avoid sexual activities for material gain and appropriate and consistent use of condoms during sexual intercourse can be enhanced significantly by the implementation of integrated RH lesson materials in a PBP. The integrated RH lesson materials in a PBP have demonstrated a significant effect in enhancing critical thinking and problem-solving skills among adolescents of both sex to address SRH problems in their day-to-day interactions. However, adolescents exposed hybrid PBP scored higher in soft skills for safe sexual behaviors across the study timelines against their counterparts in the LBP compared to those in the pure PBP against the LBP. If hybrid PBP may be adopted and implemented widely in ordinary level secondary schools to facilitate SRH learning, it might help to empower adolescents with soft skills necessary for them to make informed, reasoned, and responsible decisions over their sexual behaviours. On the other side of the coin, the integrated RH lesson material in a PBP, the hybrid PBP in particular might serve as a formal guide to teachers, health workers, and other facilitators in facilitating SRH learning among adolescents in ordinary level secondary schools. By so doing, it may help to delay the onset of sexual activities and therefore unintended teenage pregnancies, new STIs, and school dropouts, which are linked with teenage pregnancies among adolescents.

### Strengths of the study

This study explored an important issue given robust evidence, which suggests that curriculum-based sex education interventional programs or projects are potential in shaping safe sexual behavior among adolescents. The study has advocated multidisciplinary strategies by developing integrated RH lesson materials in a PBP to guide teachers and or health workers in facilitating SRH learning among adolescents in Tanzania. Needless to say, this study has managed to develop procedural prescriptions about how to develop the integrated RH lesson materials in a PBP alongside its implementation and evaluation.

The study has managed to demonstrate the mechanisms to be adhered to produce and measure outcome variables such as adolescents’ SRH knowledge, soft skills, and sexual behaviors in the Tanzanian context. The current study has addressed the methodological gap by adopting sequential mixed methods through phenomenological design and randomized controlled trials to examine the effectiveness of integrated RH lesson materials in a PBP on adolescents’ SRH knowledge, soft skills, and sexual behaviors. Many previous studies are silent on SRH matters for the full range of adolescent stages (Early, middle and late), a gap that this study addressed accordingly in addressing the increased rate of new STIs/HIV, unintended teenage pregnancies, and their associated obstetric complications, early marriages, and school dropouts among adolescents.

### The implication of the study

The findings of this study might add new knowledge on the best ways to develop, adopt and evaluate innovative SRH pedagogies in a PBP via a randomized controlled trial to enhance its facilitation among adolescents. Moreover, the findings might provide a path towards developing a sustainable, interdisciplinary, and formal SRH guideline to assist and guide teachers and or health workers during the facilitation of SRH learning among adolescents. Adolescents may also benefit from the findings of this study because they are going to be oriented on necessary soft skills to be adhered to against sexual storms and stresses in their day-to-day living.

### Limitation of the study

There were some limitations, which this study faced. Among others, one was a lack of reliable evidence on actual comprehensive sexual education lesson content delivered in schools in the absence of video recording of sessions or active observation by the evaluation team. Thus, this study was unable to verify the completeness of the content in lessons during delivery. However, there were verifiable research assistants’ reports and students’ feedback that were proxy evidence for this. Additionally, the same researchers who implemented the study, which would have led to a biased interpretation of the results, conducted the evaluation. To address the reporting bias, the developed reporting checklist was adhered to.

Desirability bias was another limitation faced by this study as the questionnaires included sensitive questions (items) that would lead the study participants to answer them in a manner they felt to be socially inappropriate. It was impossible to determine in what direction such a bias operated but, the researcher and assistants reassured the study participants of anonymity and to conceal participation. Additionally, participants were seated in a separate room from their teachers and parents, each of them sitting on a sparse single chair and desk, and were assured of their privacy, confidentiality, and security of the information they provided. This helped to minimize the mentioned bias above.

Besides, the study participants were school-going students and thus, the bias would have occurred as the interventions were conducted in the school environment and participants could have consequently been over-emphasized about the importance of new knowledge obtained in schools. Every attempt was made by the researchers and assistants to minimize the bias by using critical incidents and real-world scenarios that occur in day-to-day life to make them intellectually move from the school, classrooms, and chairs to their homes, streets, and real-life activities. Nevertheless, it is important to note that implementing curriculum-based sex education as a preventive measure intervention in schools is challenging particularly, in terms of aligning it with the schools’ semester or schedules. However, the researchers of this study fixed the research-intervention schedule after discussing and negotiating with the headmasters of the sampled secondary schools to minimize the bias.

Moreover, this study was done in Tanzanian secondary schools, and participants would differ in some sociodemographic and academic aspects including their year of study, age, gender, cultures, traditions, socio-economic status, and academic abilities. This would influence the selection, information, and recall biases. Every attempt was made to stratify and match them based on their sociodemographic characteristics and random allocation of schools into either an intervention or control group was performed. However, the outcomes of the study might be affected by the extraneous variables and therefore results need to be interpreted cautiously. Likewise, the findings of this study might not be generalized to other education levels such as kindergarten, primary, certificate, diploma, degrees, or master’s program.

Besides, the world and country (Tanzania) faced a public and epidemiological incidence of new respiratory viral infection named “Corona Virus (COVID-19) that led to the temporary closure of all schools over the country. The status affected this study especially the third-fourth phases of data collection (follow up) because they had to be extended for the schools to be reopened. The incidence of COVID-19 might have led to psychological disturbances (fear) among people perhaps including the sampled study participants that would affect their memory on the study and thus affect the intended and targeted findings. Therefore, the findings from the fourth phase need to be interpreted curiously.

## Supporting information

S1 Data (SAV)

## Abbreviations

AIDS: Acquired Immunodeficiency Syndrome
HIV: Human Immunodeficiency Virus
IRRC: Institutional Research Review Committee
ITT: Intention To Treat Analysis
LBP: Lecture-based Learning
MOEVT: Ministry of Education and Vocational Training
MoHCDGEC: Ministry of Health Community Development, Gender Elderly, and Children
PBP: Problem-based Pedagogy
PO-RALC: Permanent Secretary Regional Administration and Local Government
SAS: Statistical Analysis System
RH: Reproductive Health
SRH: Sexual and Reproductive Health
STIs: Sexual Transmitted Infections
UDOM: University of Dodoma
WHO: World Health Organization
